# *Lactobacillus salivarius* extracellular vesicles enhance gut and liver function in MAFLD

**DOI:** 10.3389/fimmu.2025.1660131

**Published:** 2025-08-28

**Authors:** Lihui Zhu, Jiwen Huang, Zhen Luo, Huaxiang Yan, Xiao Wu, Rongrong Liao

**Affiliations:** ^1^ Institute of Animal Husbandry and Veterinary Science, Shanghai Academy of Agricultural Sciences, Shanghai, China; ^2^ Shanghai Key Laboratory of Veterinary Biotechnology, School of Agriculture and Biology, Shanghai Jiao Tong University, Shanghai, China; ^3^ Key Laboratory of Agricultural Genetics and Breeding, Biotechnology Research Institute, Shanghai Academy of Agricultural Sciences, Shanghai, China

**Keywords:** extracellular vesicles, *Lactobacillus salivarius*, fatty liver, mitophagy, lipid metabolism

## Abstract

**Background:**

Metabolic dysfunction-associated fatty liver disease (MAFLD) is associated with an accumulation of fat in the liver, disruptions in lipid metabolism, and imbalances in the gut microbiome. Extracellular vesicles (EVs) derived from probiotics have emerged as potential mediators of host lipid metabolism effect. The precise mechanisms by which EVs derived from probiotics influence MAFLD are still not fully understood.

**Methods:**

We examined the therapeutic potential of EVs sourced from *Lactobacillus salivarius* SNK-6 (LsEVs) using a mouse model of MAFLD and fatty acids induced cells.

**Results:**

Oral LsEVs administration reduced weight gain, lower liver enzyme levels, and less liver fat in mice. Meanwhile, LsEVs increases the secretion of anti-inflammatory factor IL-4 in mice subjected to a high-fat diet, but inhibited the pro-inflammatory cytokine secretion in lipopolysaccharide induced gut cells. Mechanistically, LsEVs enhance liver cell mitophagy via Beclin-1 and PPAR related pathways. LsEVs also increased tight junction proteins in epithelial cells. Furthermore, LsEVs boost gut bacterial diversity in MAFLD -afflicted mice by promoting beneficial *Bacteroidota* and suppressing harmful *Desulfovibrio*.

**Conclusions:**

Our research established a foundation for the future use of LsEVs in treating MAFLD and provided novel insights into the mechanisms of lipid metabolism influenced by EVs derived from probiotics in the context of MAFLD.

## Graphical Abstract

LsEVs derived from L. salivarius SNK-6 enhanced gut and liver function in MAFLD by regulating gut microbiota composition, enhancing cell mitophagy, and suppressing lipid deposition.

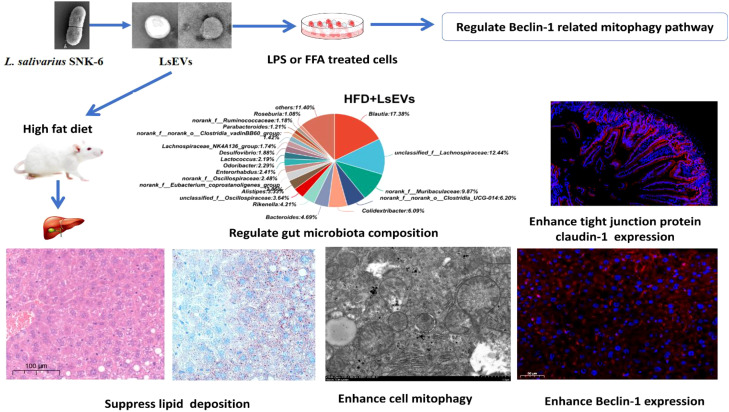

## 1 Introduction

Metabolic dysfunction-associated fatty liver disease (MAFLD), previously known as nonalcoholic fatty liver disease (NAFLD), affects approximately 25% of the global population. This condition exhibits the highest prevalence in the Middle East (31%) and South America (28%), and is pathologically characterized by hepatic steatosis not induced by alcohol consumption (1, 2). The condition may also advance from basic fat accumulation to more significant inflammation and fibrosis (3, 4). At present, there are no medications authorized for the treatment of MAFLD; therefore, management primarily focuses on lifestyle modifications such as dietary changes and weight reduction (5). The gut microbiota significantly influences MAFLD development by affecting energy metabolism, inflammation, and gut permeability (6). Recent studies suggest that probiotics, particularly lactic acid bacteria, can enhance liver function by altering the intestinal microbiota, reducing inflammation, and enhancing the intestinal barrier (7, 8). For example, *Lactobacillus plantarum* improves liver function, reduces fat accumulation, and lowers inflammation in rats (9). Additionally, *Lactobacillus acidophilus* releases valeric acid, showing anti-cancer effects in mice (10). Probiotics, like *Lactobacillus plantarum* ZJUIDS14, may prevent MAFLD by improving intestinal barrier integrity, modulating gut microbiota, and enhancing mitochondrial function (11). However, the precise way in which probiotics address MAFLD remains unclear, necessitating further research to explore this issue. Our past investigations revealed that *Lactobacillus salivarius* SNK-6 (*L. salivarius* SNK-6) can significantly prevent liver fat buildup in laying hens (12), however, the detail mechanism is still unclear.

Cells or bacteria release extracellular vesicles (EVs), which are membrane-like structures crucial for cell communication and substance exchange (13). Probiotic-derived EVs have shown potential therapeutic effects, such as anticancer, anti-inflammatory, antioxidant, and antidiabetic properties. They can reduce inflammatory factor expression and enhance host defense gene activity, thereby strengthening the immune response (14, 15). For instance, *Akkermansia muciniphila*-derived EVs have been shown to lower tumor necrosis factor α (TNF-α) and interleukin 6 (IL-6) production and reduce fat deposition caused by a high-fat diet (14). Probiotic EVs, such as those from *Lactobacillus plantarum*, can boost the immune system by promoting polarization of M2 macrophages and triggering an anti-inflammatory response in human skin (15). A recent study reported that *L. paracasei*-derived EVs alleviate neuroinflammation in rats with high ammonia levels and repair the TNFα-TNFR1-S1PR2-IL-1β-CCL2-BDNF-TrkB pathway in hyperammonemic rats (16). Previously, we extracted EVs from *L. salivarius* SNK-6 (LsEVs) and found that they can be absorbed by host cells and trigger an anti-inflammatory response, indicating their potential as a safe treatment for fatty liver (17). Here, we investigated how LsEVs inhibit fatty liver. Our findings reveal that LsEVs reduce fat deposition by enhancing mitophagy, maintaining intestinal barrier integrity, and regulating the gut microbiome in MAFLD caused by a high-fat diet (HFD), offering new insights for fatty liver treatment.

## 2 Materials and methods

### 2.1 LsEVs isolation

A single colony of *L. salivarius* SNK-6 was inoculated into 1000 mL of MRS medium. The culture was shaken at 220 rpm and incubated under anaerobic conditions at 41 °C for 24 h to achieve a final concentration of approximately 2 × 10^8^ CFU/mL of the *L. salivarius* SNK-6 mixed solution. LsEVs were obtained from the culture supernatant at 4 °C following previously outlined technique (17). Briefly, to remove cell debris and aggregates, the bacterial culture medium underwent centrifugation at a low speed (12,000 × g for 20 minutes). Following this, the supernatant was collected and filtered with 0.45 μm membrane to remove larger molecules. Then, the filtrate was transferred into a 15 mL 3-kDa ultrafiltration system and centrifuged for 30 minutes at an average speed of 3,000 × g. Finally, total exosome isolation reagent (from cell culture media, Invitrogen, USA) was added to the retained filtrate overnight, and the EVs were separated at 12,000 × g for 1 h, according to the manufacturer’s guidelines. The EVs were then resuspended in 1mL RNA-free water and kept at -80 °C. High concentrations of LsEVs were achieved by merging LsEVs from the culture media collected over four different periods and concentrating them with a 3-kDa ultrafiltration system (Millipore, MA, USA). According to our prior nanoparticle tracking analysis, LsEVs were mostly between 100 and 250 nm in diameter, with the largest reaching 236 nm (17). The BCA protein assay kit (Sangon Biotech, Shanghai, China) is used to determine the protein concentration of LsEVs. Finally, the purified LsEVs were identified by transmission electron microscopy (JEOL, Tokyo, Japan) and details are described below.

### 2.2 Animal experiments

Forty 35-day-old male BALB/c mice were selected and classified into four groups: a normal diet group (conventional diet + 100 μL PBS), a high-fat group (HFD + 100 μL of PBS), an SNK-6 treated group (HFD + 100 μL of 1 × 10^8^ CFU/mL *L. salivarius* SNK-6), and an LsEVs treated group (HFD + 100 μL of 10 μg LsEVs). The mice were provided with a standard diet and kept in an environment with a 12 h light and dark cycle at temperatures ranging from 21 to 25 °C, with humidity levels maintained at 55 ± 10%. Subsequently, mice from the normal diet were treated with conventional diet, while mice in the HFD, SNK-6, and LsEVs treated group were free consumption of a high-fat feed for two weeks, after which, from the 56-day-old onward, each group received gavages of 100 μL PBS, *L. salivarius* SNK-6, or LsEVs. After 12 weeks (140-day-old), the mice were euthanized, and blood was drawn from their eyes. The serum was obtained by centrifuging the blood at 4,000 rpm for a duration of 10 minutes, after which it was stored at -80 °C for future analysis. Liver tissue was gathered and aseptically frozen at -80 °C. The animal studies adhered to the guidelines and regulations provided by the Ethics and Animal Welfare Committee of the Shanghai Academy of Agricultural Sciences (approval number: SAASPZ0521022). The animal sample size was determined through a *post hoc* analysis of the achieved power using G*Power 3.1.9.7 (Heinrich Heine Universität, Düsseldorf, Germany). With a significance level (α) set at 0.05, an effect size (d) of 1.5, and a sample size of 10 animals per group, we attained a widely recognized statistical power (1−β) of 0.92.

### 2.3 Biochemical analysis

To assess the effectiveness of different substances, we acquired commercial kits for alanine aminotransferase (ALT), aspartate aminotransferase (AST), total cholesterol (T-CHO), and triglycerides (TG) from Nanjing Jiancheng (Nanjing, China). The serum concentrations of TNF-α, IL-2, IL-6, and IL-4 were determined using enzyme linkedimmunosorbent assay kits following the manufacturer’s instructions (Jingmei Bio, Jiangsu, China).

### 2.4 Hematoxylin and eosin staining

The livers and jejunum samples were preserved in 4% paraformaldehyde, subjected to dehydration through rising concentrations of ethanol, cleared using xylene, and then encapsulated in paraffin. They were sliced into 5 μm sections, processed with xylene, absolute ethanol, and decreasing ethanol concentrations, dewaxed, rinsed with double-distilled water, and dried. Subsequently, the sections were stained with alkaline hematoxylin and 0.5% eosin, followed by scanning with an Olympus VS200 (OLYMPUS Corporation, Japan) slide scanner.

### 2.5 Oil Red O staining

Livers were initially preserved with 4% paraformaldehyde, then dehydrated in 20% sucrose, and embedded in Tissue Tek OCT (Sakura, Netherlands). They were quickly frozen and sliced into 8 μm sections. Subsequently, Oil Red O staining was performed using Solarbio’s kit (Beijing, China), following the instructions. Image Pro Plus 6.0 software (Media Cybernetics, Bethesda, MD, USA) was used for analyzing positive staining and slice area.

### 2.6 Transmission electron microscopy

TEM was performed according to established protocols (18). Briefly, purified LsEVs and livers were preserved overnight in a 2.5% glutaraldehyde and 0.05M sodium phosphate buffer solution at pH 7.2. Following the washing of samples in 0.1M diethyl carbonate buffer combined with saline, the next steps involved dehydrating the samples using ethanol, embedding them in epoxy resin, and slicing them into ultra-thin sections measuring 50 to 100 nm. These sections were then placed onto copper grids, subjected to staining with uranyl acetate and lead citrate, and observed under a Hitachi H7700 transmission electron microscope (Tokyo, Japan).

### 2.7 Immunofluorescence staining analysis

Liver tissues embedded in paraffin were cut into sections of 5 μm thickness and subsequently processed using relevant primary Beclin-1 antibodies (Servicebio, Wuhan, China; GB115741). Concurrently, jejunum sections were incubated with claudin-1 antibody (Servicebio, GB12032). The sections underwent incubation in a blocking solution before being incubated with fluorescent isothiocyanate-labeled secondary antibodies for 30 min at 37 °C. Following washes with PBS, the sections were stained with DAPI for 10 min. Finally, the sections were mounted with neutral resin and examined under a fluorescence microscope.

### 2.8 Cell culture

The NCTC1469 human hepatocyte line (CCL-227, ATCC) was cultured in high-glucose DMEM (Invitrogen, Thermo Fisher Scientific, Inc.), which was enriched with 10% horse serum, under conditions of 37 °C in a 5% CO_2_/95% air environment. To verify the protective effect of LsEVs on fatty acids (FFA) challenged liver cells, the NCTC1469 cells were seeded in 12-well plates (approximate 2×10^5^ cells per well) and treated with 1 nM FFA (oleic acid: palmitic acid, 2:1) for 12 h. Following this, 20 nM LsEVs were introduced into the culture media and incubated again at 37 °C for 24 h. Meanwhile, cells treated with PBS in the absence of FFA and LsEVs was used as negative control. Cells were gathered for RT-PCR and western blot analysis.

To verify the protective effect of LsEVs on lipopolysaccharide (LPS) challenged intestinal epithelial cells, human intestinal epithelial Caco-2 cells, sourced from ATCC, were cultivated in DMEM (Invitrogen) along with 10% FBS (Invitrogen). Caco-2 cells (5 × 10^5^ cells/ml) were treated with PBS, or LPS (1 μg/ml), or LPS (1 μg/ml) + LsEVs (10nM) for 24 h. Cell supernatant and cellular samples were then harvested for subsequent analysis. The appropriate amount of LsEVs to be added to cells based on cell viability determined by Cell counting kit-8 (CCK-8, Beyotime, Shanghai, China). Details were shown in **Supplementary Figure S1**.

### 2.9 Quantitative Real-Time PCR

RNA extraction was conducted using Trizol Reagent (TIANGEN Biotech, Beijing, China). Subsequent to this, 1 µg of RNA underwent reverse transcription employing Prime Script™ RT Master Mix (Perfect Real Time) (Applied Biosystems, Waltham, MA, USA). The quantitative analysis of genes was conducted using Power Up SYBR Green MasterMix on a Step One Plus Real-Time PCR Instrument (Applied Biosystems). GAPDH and β-actin served as the housekeeping gene. The primers are listed in **Supplementary Table S1**.

### 2.10 Western blot analysis

Equal volumes of protein samples were carefully loaded onto polyacrylamide gels, subjected to electrophoresis, and transferred onto PVDF membranes (Thermo Fisher Scientific). To detect specific proteins, various primary antibodies were used, such as those targeting LC3 (GB113801), peroxisome proliferator-activated receptor γ (PPARγ, GB11164), sterol regulatory element-binding transcription factor 1 (SREBP1, GB11524), Beclin-1 (GB112053), claudin-1 (GB112543), and β-actin (GB15003), all sourced from Servicebio (Wuhan Servicebio Technology Co., Ltd., Wuhan, China). The presence and quantity of the target proteins were analyzed using a Super ECL detection reagent (Yeasen, Shanghai, China). The resulting bands were then quantified for analysis utilizing Image J software (NIH, Bethesda, MD).

### 2.11 16S rRNA gene sequencing

Genomic DNA was obtained from fecal samples of normal diet, HFD, and LsEVs treated mice. The amplification of the bacterial 16S rRNA genes was conducted on the V3–V4 region using the primer pairs 338F (5’-ACTCCTACGGGAGGCAGCAG-3’) and 806R (5’-GGACTACHVGGGTWTCTAAT-3’). This process utilized a T100 Thermal Cycler PCR device (BIO-RAD, USA). Following purification, the amplicons were combined in equal molar concentrations and then subjected to paired-end sequencing on an Illumina Nextseq2000 platform (Illumina, San Diego, USA), in accordance with the standard protocols specified by Majorbio Bio-Pharm Technology Co. Ltd. (Shanghai, China).

Following demultiplexing, quality filtering of the resulting sequences was performed using fastp (version 0.19.6) (https://github.com/OpenGene/fastp) and the merging process was conducted with FLASH (version 1.2.11) (http://www.cbcb.umd.edu/software/flash). Subsequently, high-quality sequences were subjected to de-noising using the DADA2 plugin within the Qiime2 (version 2020.2) framework. This process utilized the recommended settings that ensure single nucleotide resolution by analyzing error profiles within the samples. To reduce the impact of sequencing depth on the measures of alpha and beta diversity, the sequence count from each sample was rarefied to 20,000, which resulted in an average Good’s coverage of 97.90%. The functional capabilities of the microbial communities were predicted employing PICRUSt, leveraging the high-quality sequences. LEfSe was applied to identify bacterial species that contribute to differences among the experimental groups.

### 2.12 Statistical analysis

Data underwent a one-way ANOVA analysis, subsequently followed by Tukey’s test for multiple comparisons, utilizing SPSS 13.0 software (SPSS, Chicago, IL). Statistical significance between the two mean values was determined by an adjusted *P*-value of less than 0.05.

## 3 Results

### 3.1 LsEVs inhibit inflammatory response of HFD-fed mice

Recently, we discovered that *L. salivarius* SNK-6 exhibits a protective effect against fatty liver in laying hens. In our previous study, we applied a fixed concentration of *L. salivarius* SNK-6 at 1 × 10^8^ CFU/kg (12). To investigate the effect of LsEVs on MAFLD, mice were subjected to daily oral gavage administration (100 µL) of either *L. salivarius* SNK-6 or LsEVs for a duration of 84 days. The dose of LsEVs used in this study was determined based on the findings reported by Hao et al. (19) and Hu et al. (20).

**Figure 1:**
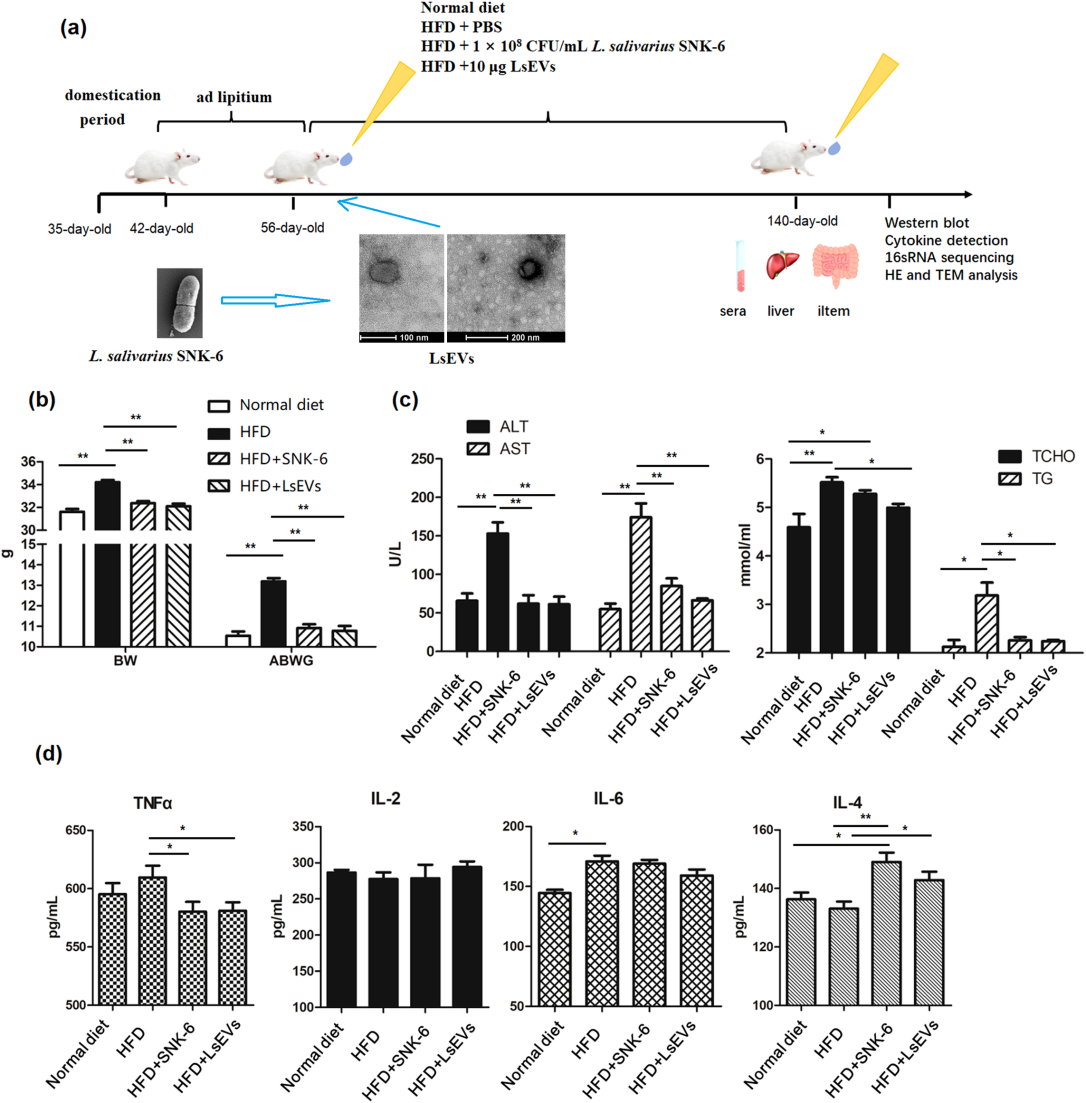
LsEVs treatment reduced body weight gain, enhanced the secretion of anti-inflammatory cytokine in MAFLD mice. **(a)** Experimental design; **(b)** BW changes after LsEVs treatment. **(c)** The treatment with LsEVs reduces the activities of ALT and AST, along with lowering the levels of T-CHO and TG. **(d)** LsEVs therapy increases the secretion of IL-4 in mice subjected to HFD. BW, body weight; ABWG, average body weight gain; TG, triglyceride; T-CHO, total cholesterol; ALT, alanine aminotransferase; AST, aspartate aminotransferase; HFD, high fat diet; SNK-6, *L. salivarius* SNK-6; LsEVs, *L. salivarius* SNK-6-derived extracellular vesicles. * *P* < 0.05 and ** *P* < 0.01 (*N* = 9).

Here, we found that *L. salivarius* SNK-6 and its secreted LsEVs were supplemented to the HFD diet (**Figure 1a**) to evaluate the function of LsEVs on fatty liver prevention. As shown in **Figure 1b**, we found a significant increase in the body weight on HFD mice, when comparing to the normal diet treated mice (*P <*0.05). Oral administration of *L. salivarius* SNK-6 and LsEVs significantly reduced weight gain of mice, comparing with the HFD treated mice (*P <*0.05). In comparison with the control groups, HFD led to a significant rise in serum T-CHO and TG, potentially contributing to the development of fatty liver (**Figure 1c**). Additionally, the activities of AST and ALT were markedly higher in the HFD groups compared to the other three groups. When *L. salivarius* SNK-6 and LsEVs were administered orally, a noticeable decrease in TNF-α levels was observed; however, no significant reduction in the pro-inflammatory factors IL-2 and IL-6 occurred in mice treated with *L. salivarius* SNK-6 and LsEVs when compared to those treated with the HFD. Conversely, a significant increase in the anti-inflammatory factor IL-4 (*P*<0.05) was noted in mice receiving *L. salivarius* SNK-6 and LsEVs (**Figure 1d**).

### 3.2 LsEVs attenuate fat deposition of HFD-fed mice

**Figure 2:**
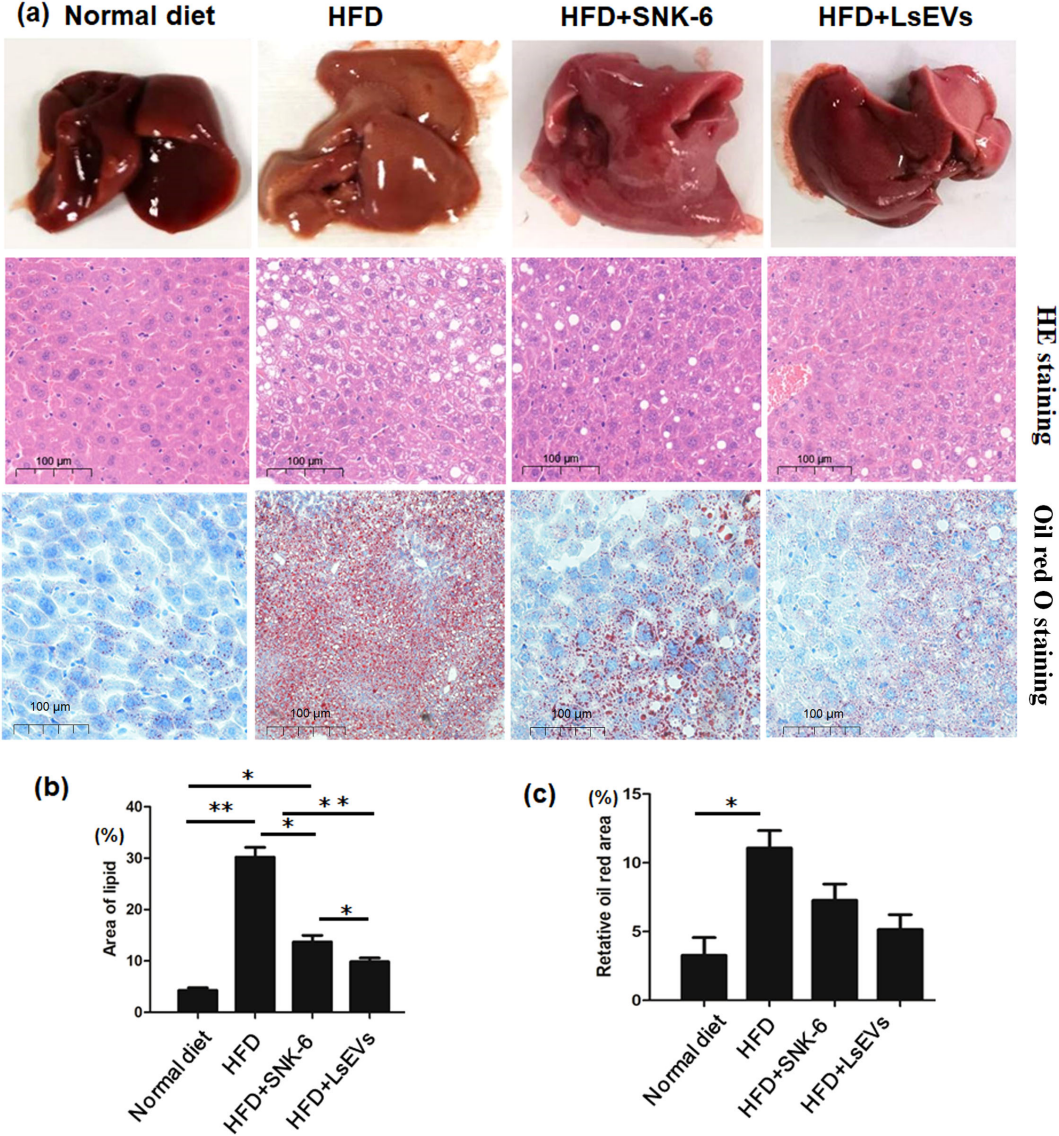
Histological assessment. **(a)** Representative images of H&E-stained and Oil Red O Staining slides **(b)** Quantification of injury area analyzed by H&E-stained and **(c)** Oil Red O staining in liver. The data is presented as the mean ± SEM. *P < 0.05, **P < 0.01, *N* = 9 each group. HFD, high fat diet; SNK-6, *L. salivarius* SNK-6; LsEVs, *L. salivarius* SNK-6-derived extracellular vesicles.

As depicted in **Figure 2**, mice in HFD group have livers with a brownish-yellow color, fragile and soft texture, and large vesicular steatosis with many fat vacuoles. In contrast, mice treated with *L. salivarius* SNK-6 and LsEVs have somewhat yellow livers with a smooth, elastic texture and fewer fat vacuoles. Oil Red O staining reveals significant fat accumulation in the HFD group, with many irregular red lipid droplets, whereas the control group shows minimal droplets. The LsEVs treatment group has some red lipid droplets, but significantly fewer than the HFD group (**Figure 2a**). In comparison to the HFD group, mice that received treatment with *L. salivarius* SNK-6 and LsEVs exhibited a remarkable decrease in liver lipid droplet counts (*P*<0.05). Notably, the group administered LsEVs demonstrated a significant reduction in hepatic lipid droplets (*P*<0.05), suggesting a reduction in lipid buildup and a more substantial improvement in metabolic disorders related to lipid accumulation induced by the high-fat diet (**Figures 2b, c**).

### 3.3 LsEVs activated autophagy of HFD-fed mice

**Figure 3:**
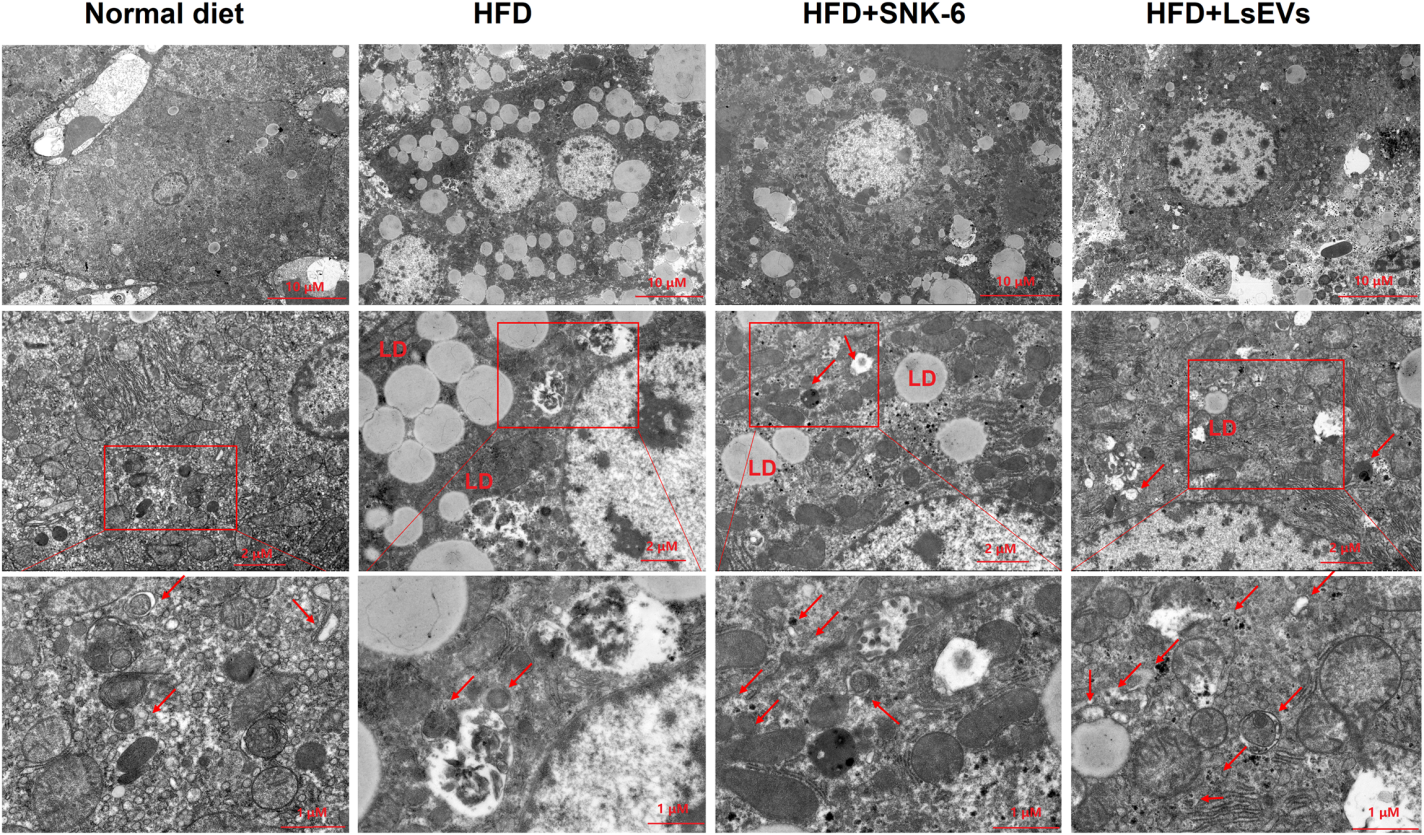
Transmission electron microscopy analysis. Autophagosomes are shown by red. LD: lipid droplet; HFD, high fat diet; SNK-6, *L. salivarius* SNK-6; LsEVs, *L. salivarius* SNK-6-derived extracellular vesicles.

TEM was utilized to observe autophagosomes, mitophagosomes, and autophagic lysosomes. In **Figure 3**, no obvious lipid droplets were detected in the liver cells of the control group mice. The integrity of the cellular structure was intact, with cytoplasmic density appearing normal. The mitochondrial structure was also preserved, exhibiting a dense and regular arrangement. Conversely, the liver cells from the HFD group exhibited a higher quantity of dispersed lipid droplets of different sizes, along with structural impairments, sparse cytoplasmic swelling, and notable mitochondrial enlargement. In contrast to the HFD mice, there was a considerable decrease in the number of lipid droplets within the liver cells following treatment with *L. salivarius* SNK-6 and LsEVs, and autophagosomes developed in the mouse liver. The number of autophagosomes and autophagic lysosomes increased, and mitochondrial damage was reduced (**Figure 3**). An elevation in autophagy was noted in the mice of *L. salivarius* SNK-6 and LsEVs treated group, when compared to those on HFD mice.

**Figure 4:**
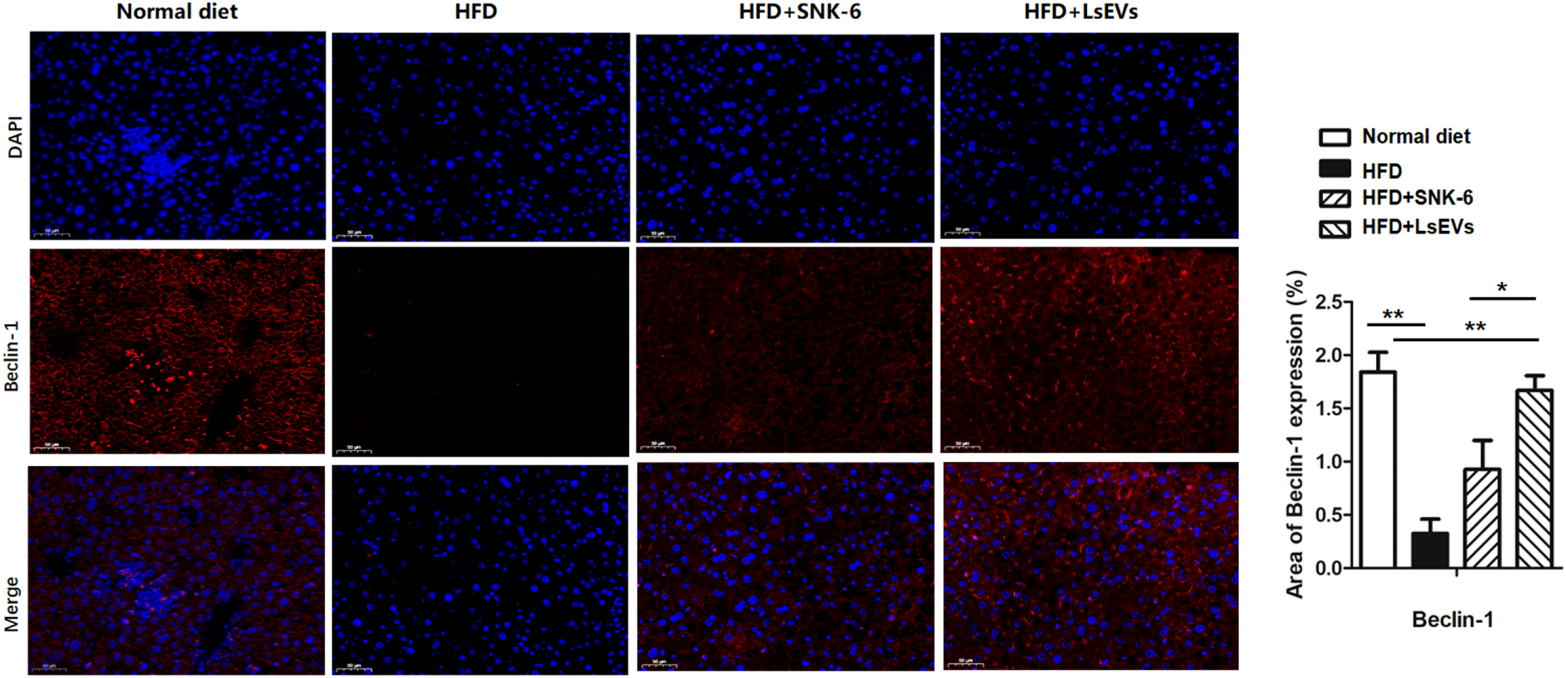
LsEVs treatment enhanced autophagic activity in HFD treated mice. **(a)** Representative immunofluorescence staining images of liver in HFD treated mice (*N* =6). Red Beclin-1, Blue: nucleus. Scale bar: 20 μm. **(b)** Fluorescence intensity analysis of mitophagy marker proteins Beclin-1 in the HFD treated mice. Results are presented as mean ± SEM. Annotations of * *P* < 0.05 and ** *P* < 0.01 indicate significance. HFD, high fat diet; SNK-6, *L. salivarius* SNK-6; LsEVs, *L. salivarius* SNK-6-derived extracellular vesicles.

Immunofluorescence analysis revealed that, Beclin1 proteins were located near the nuclei as depicted in the control group (**Figure 4a**). In contrast, the HFD group displayed a marked reduction in Beclin1expression, while a partial recovery was noted in the groups treated with *L. salivarius* SNK-6 and LsEVs groups (**Figure 4b**).

### 3.4 LsEVs regulated Beclin-1 mitophagy related pathway of HFD-fed mice

**Figure 5:**
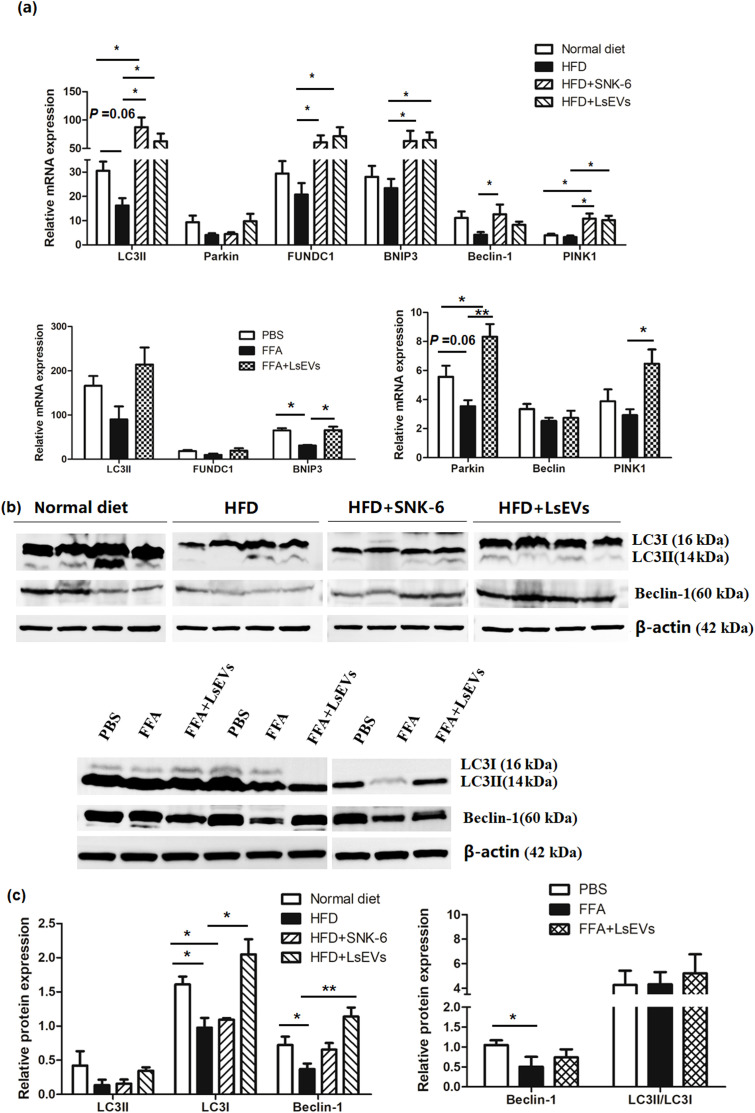
LsEVs treatment enhances the expression of mitophagy related pathway markers**(a)** Quantitative RT–PCR analysis of mitophagy related genes in LsEVs treated mice and FFA induced liver cells. **(b)** Western blot for LC3 and Beclin-1 on protein lysates prepared from the livers of LsEVs treated mice and FFA induced liver cells. The graphs illustrate the quantification of band intensities from western blot analyses, with data compiled from four separate experiments involving three mice in each group, or from three independent experiments utilizing liver cells. An unpaired two-tailed t-test was employed to evaluate the data. *P < 0.05 and **P < 0.001 was significant. **(c)** Quantitative densitometry analysis of mitophagy proteins. HFD, high fat diet; SNK-6, *L. salivarius* SNK-6; LsEVs, *L. salivarius* SNK-6-derived extracellular vesicles; FUNDC1, FUN14 domain-containing protein 1; PINK1, PTEN induced putative kinase 1.

Genes crucial for the autophagy-related pathway were analyzed using RT-qPCR (**Figure 5a**). It was noted that the mitophagy-related genes such as *FUN14* domain-containing protein 1 (*FUNDC1*), *BNIP3*, *LC3-Ⅱ*, *Beclin1*, and *PTEN* induced putative kinase 1 (*PINK1*) showed significant increase in the livers of HFD mice that received *L. salivarius* SNK-6 (*P*<0.05) and LsEVs (*P*<0.01), in comparison to HFD mice. Furthermore, a marked enhancement in the mRNA levels of *Parkin* and *PINK1* in the LsEVs treated cells. In contrast, the level of *LC3-Ⅱ* was lower in the HFD mice compared to the control group. Additionally, a reduction was observed in the mRNA levels of *BNIP3* and *Parkin* in the cells treated with FFA. The expression of mitophagy-related proteins LC3 and Beclin1 were increased in the LsEVs treated mice compared to the HFD group (**Figures 5b, c**).

### 3.5 LsEVs regulated lipid metabolism in HFD-fed mice

**Figure 6:**
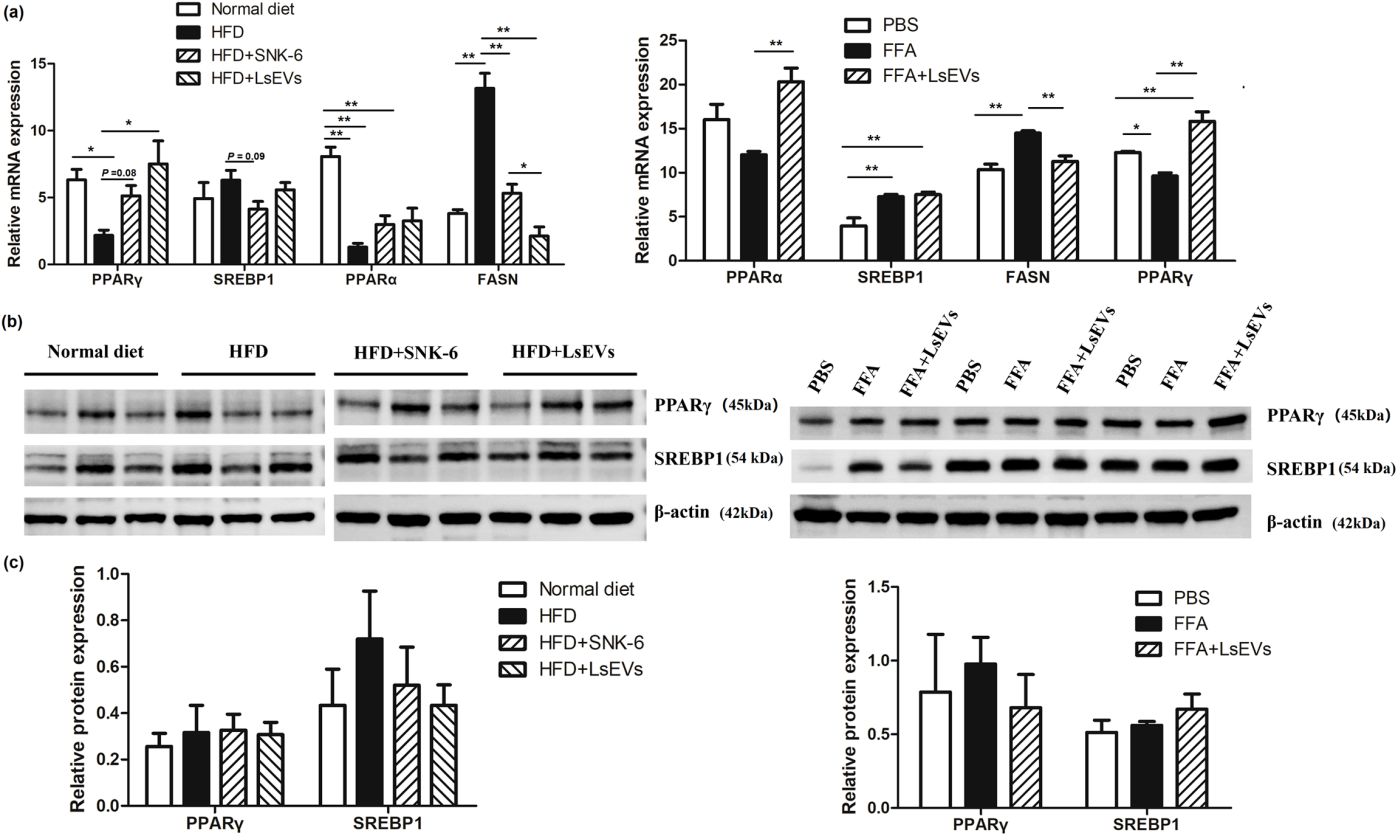
Treatment with LsEVs modulates the expression of markers associated with lipid metabolism. **(a)** Quantitative RT–PCR was conducted to assess the expression of genes related to lipid metabolism in mice treated with LsEVs and in liver cells induced by FFA. **(b)**Western blot for PPARγ and SREBP1 in protein extracts derived from the livers of LsEVs treated mice and FFA induced liver cells. The graphs illustrate the measurement of band intensities from western blot analyses, along with data aggregated from three separate experiments involving either four mice in each group or liver cells from three independent trials. *P < 0.05 and **P < 0.01 was significant. **(c)** Quantitative densitometry analysis of lipid metabolism related proteins. HFD, high fat diet; SNK-6, *L. salivarius* SNK-6; LsEVs, *L. salivarius* SNK-6-derived extracellular vesicles; SREBP1, sterol regulatory element-binding transcription factor 1; FASN, fatty acid synthase; PPAR, peroxisome proliferator-activated receptor.

We conducted additional analyses on the mRNA expression levels of genes related to lipid metabolism, which include the fatty acid oxidation genes *PPARα* and *PPARγ*, alongside genes linked to fat synthesis, like fatty acid synthase (*FASN*) and *SREBP1* (**Figure 6a**). A notable rise in *PPARα* and *PPARγ* levels was detected in mice treated with LsEVs when compared to those subjected to HFD, whereas the mRNA expression of *FASN* showed a reduction (*P* < 0.05). In FFA-treated cells, reduced levels of *PPARα* and *PPARγ* were noted, alongside increased expression of *FASN*; however, LsEVs reversed the expression of these genes. Notably, no significant differences were detected in protein levels of PPARγ and SREBP1 in the *in vivo* and *in vitro* models, although a decreasing trend in SREBP1 protein levels was noted in LsEVs-treated mice (**Figures 6b, c**).

### 3.6 LsEVs alleviate MAFLD by protecting gut barrier integrity

**Figure 7:**
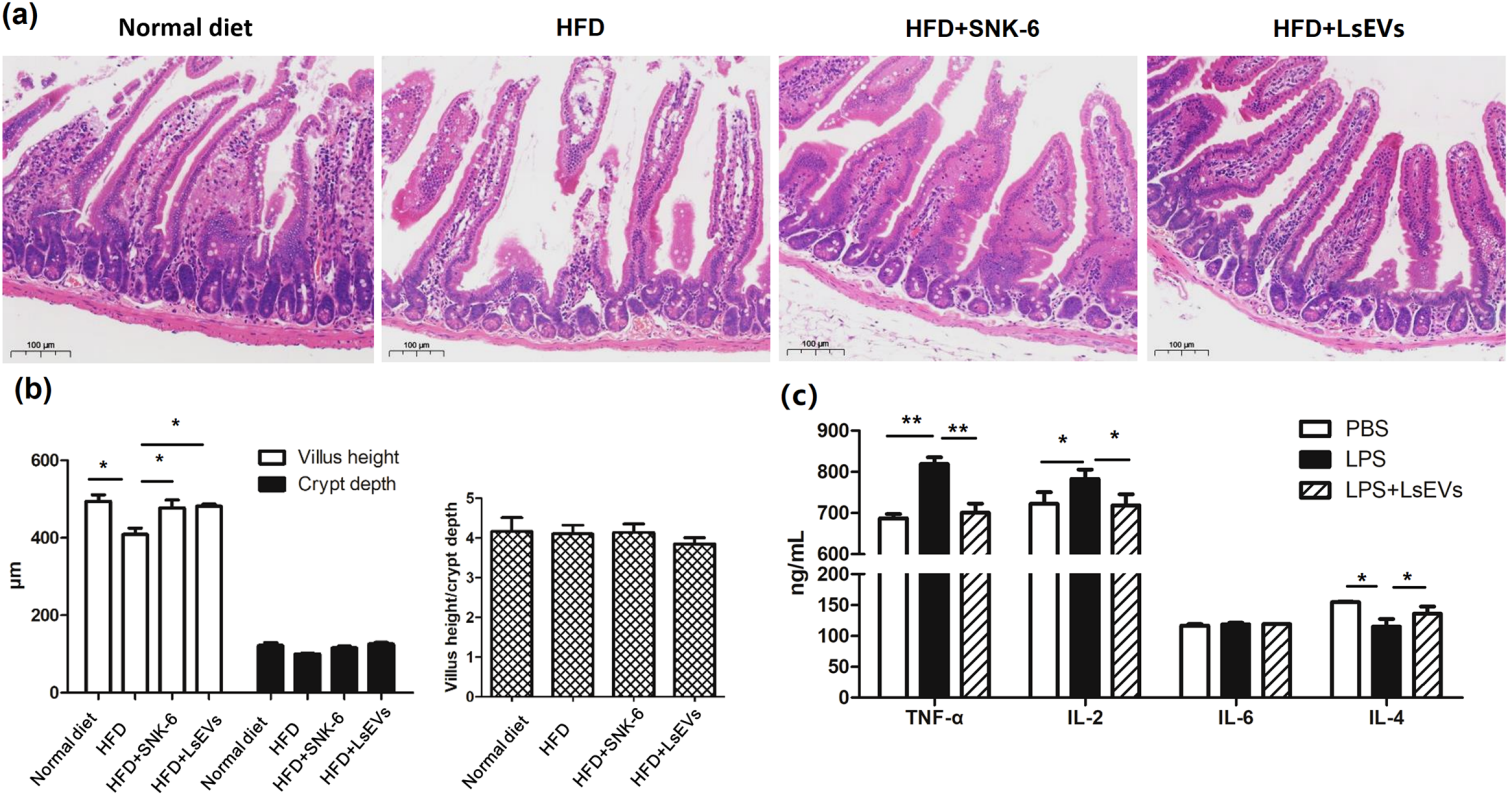
Oral administration of LsEVs mitigated the intestinal damage caused by HFD in mice. **(a)** HE staining analysis. **(b)** Impact of the oral administration of LsEVs on the height of the villi, depth of the crypts, and the villus height to crypt depth ratio (VH/CD) in the jejunum of mice challenged with HFD. **(c)** LsEVs regulated inflammatory cytokine secretion in LPS induced gut cells. HFD, high fat diet; SNK-6, *L. salivarius* SNK-6; LsEVs, *L. salivarius* SNK-6-derived extracellular vesicles; LPS, lipopolysaccharide. Annotations of * *P* < 0.05 and ** *P* < 0.01 indicate significance.

Research has shown that the functionality of the intestinal barrier is impaired in both mice and individuals suffering from fatty liver disease. In order to explore the effects of LsEVs on the intestinal barrier in mice exposed to HFD, refer to **Figure 7** for further details, we observed that in the normal mice, the structure of the intestinal mucosa layers was intact, with villi arranged in an orderly fashion and epithelial cells exhibiting a tall columnar shape. In contrast, the HFD group displayed several pathological changes, including villi atrophy, rupture defects, necrosis and shedding of mucosal epithelial cells, as well as separation of the epithelial layer from the lamina propria in the jejunum. Following treatment with *L. salivarius* SNK-6 and LsEVs, the morphology of the jejunal villi in these mice showed slight improvement, characterized by mild defects and an intact intestinal mucosal epithelium exhibiting normal morphology. Meanwhile, we used LPS to induce the stress model of gut cells. We found that LsEVs obviously reduced the contents of TNF-α and IL-2, although no significant reduction of IL-6 was found in the LsEVs treated cells, when comparing to the LPS treated cells. Meanwhile, significant increase in the content of IL-4 (*P*<0.05) was observed in the LsEVs treated cells (**Figure 7c**).

**Figure 8:**
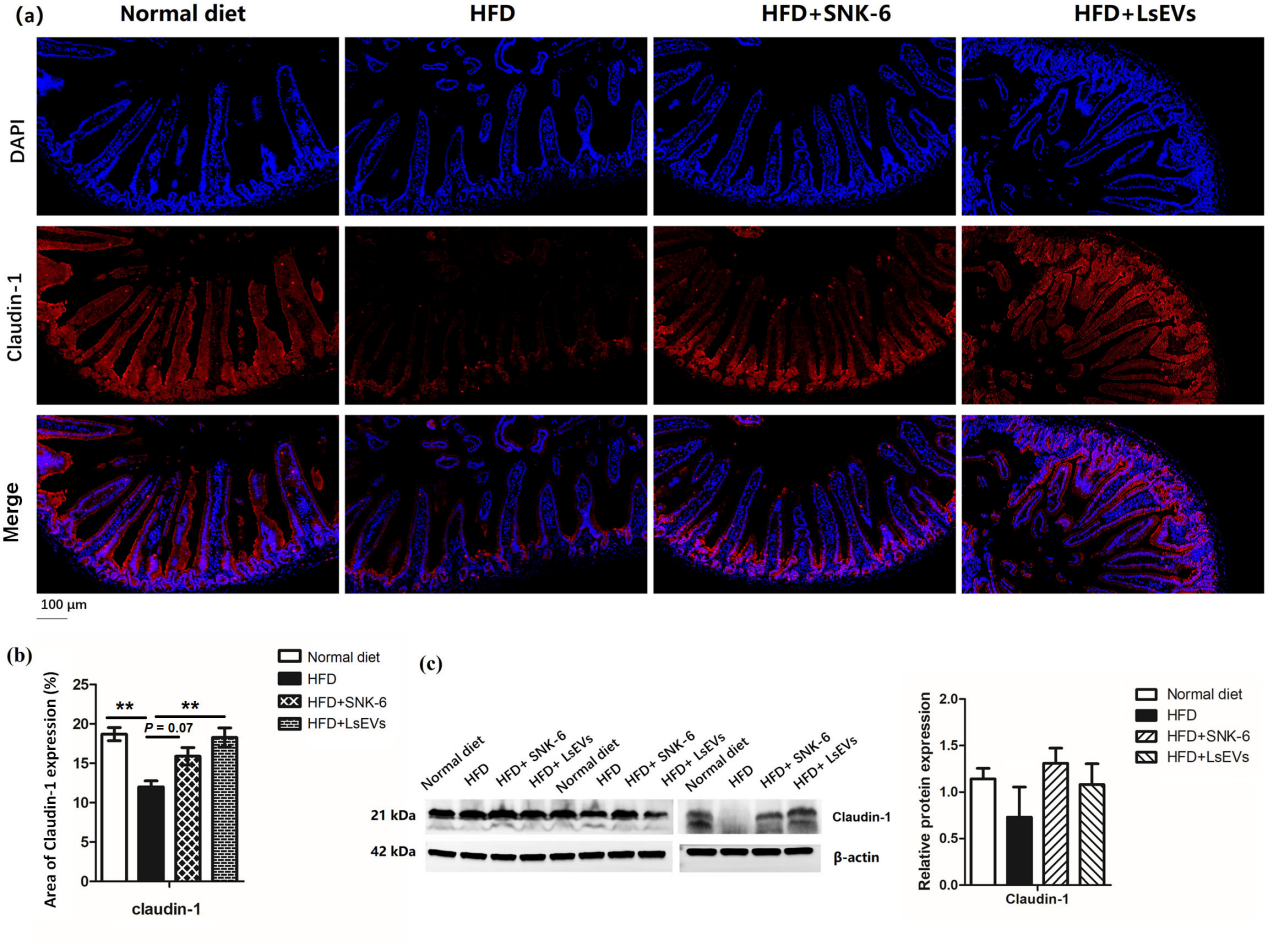
Effect of pretreatment with LsEVs on the expression levels of tight junction protein in the jejunum of mice subjected to HFD. **(a)** Representative image and **(b)** quantitative analysis of immunofluorescence staining. **(c)** Western blot for Claudin-1 on protein lysates prepared from the jejunum of mice. HFD, high fat diet; SNK-6, *L. salivarius* SNK-6; LsEVs, *L. salivarius* SNK-6-derived extracellular vesicles. Annotations of * *P* < 0.05 and ** *P* < 0.01 indicate significance.

Immunofluorescence staining was employed to observe the distribution of Claduin-1 on the surface of intestinal mucosal epithelial cells, which was consistent with the observed expression of this tight junction protein. In the HFD mice, the arrangement of Claduin-1 protein appeared loose and discontinuous, accompanied by a decrease in fluorescence intensity. Following treatment with *L. salivarius* SNK-6 and LsEVs, an increase in the fluorescence intensity of Claduin-1 protein was noted, particularly in the LsEVs treatment group, where levels approached those of the control group (**Figures 8a, b**). In the HFD-induced fatty liver mouse model, the protein level of Claudin-1 was significantly reduced, suggesting that the HFD may have disrupted intestinal barrier function (**Figure 8c**).

### 3.7 LsEVs altered bacterial communities in the ceca from mice fed HFD

**Figure 9:**
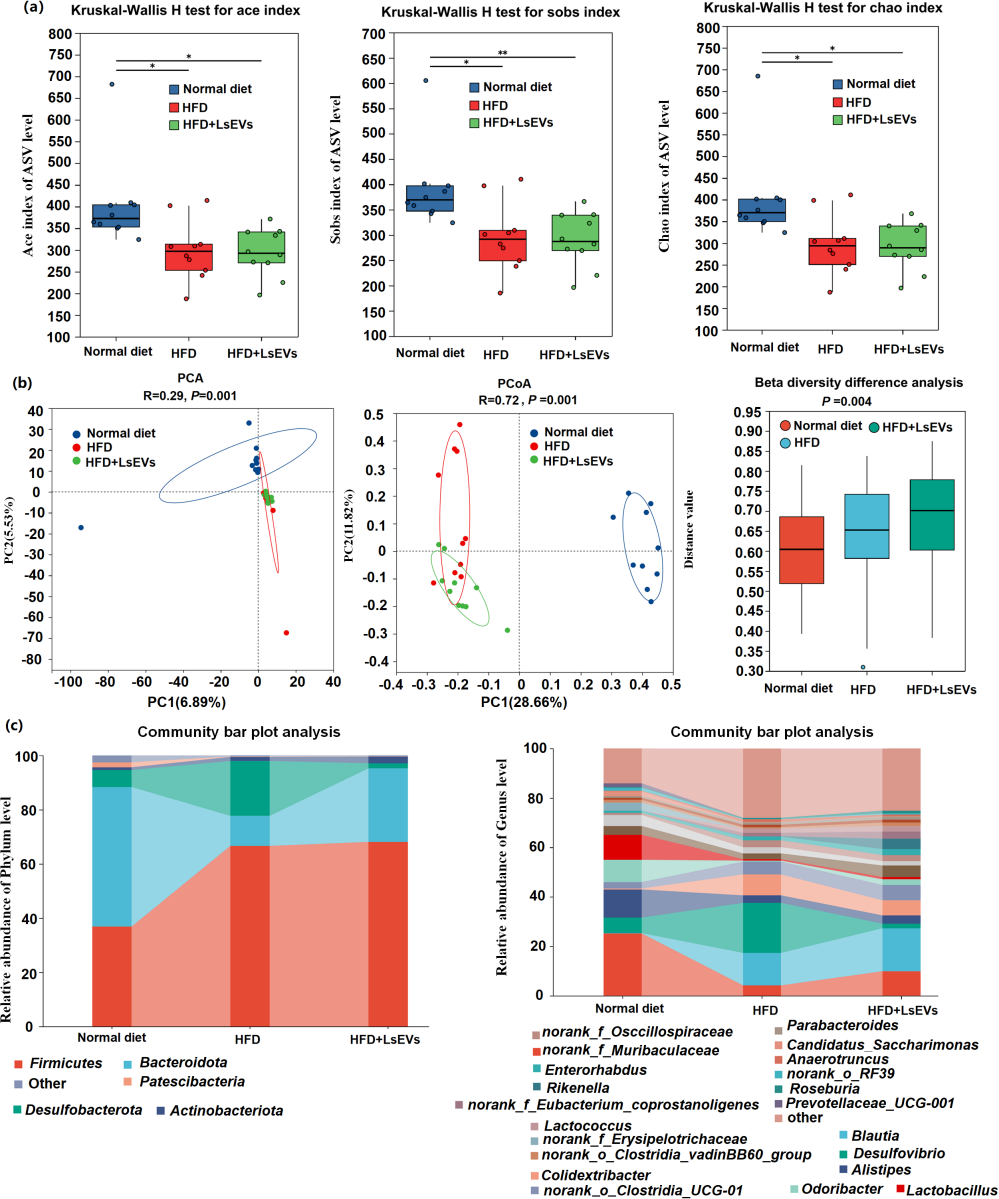
Investigation of the composition of microbial diversity in mice utilizing 16S rRNA sequencing technology. **(a)** Alpha diversity **(b)** Beta diversity of the intestinal microorganisms; Principal component analysis (PCA) and Principal coordinate analysis (PCoA) was conducted based on the Bray-Curtis distance at the ASV level. **(c)** Examination of microbial abundance in cera rabbits regarding phylum classification and **(d)** genera categorization. HFD, high fat diet; SNK-6, *L. salivarius* SNK-6; LsEVs, *L. salivarius* SNK-6-derived extracellular vesicles. Annotations of * *P* < 0.05 and ** *P* < 0.01 indicate significance.

In light of the recognized relationship between nutrition and the composition of gut microbiota, we examined how LsEVs influence the bacterial populations in the ceca of mice fed a high-fat diet. The results showed that, ace index, chao index, and sobs index of the alpha diversity were significantly changed among the normal, HFD, and LsEVs treated mice (**Figure 9a**). The beta diversity index served as a tool to assess the differences in diversity among the samples (**Figure 9b**). Analyzing the data through principal coordinate analysis (PCoA), along with principal component analysis (PCA), demonstrated a significant alteration in the microbial composition of mice subjected to HFD and LsEVs treatment in contrast to those on a normal diet.

**Figure 10:**
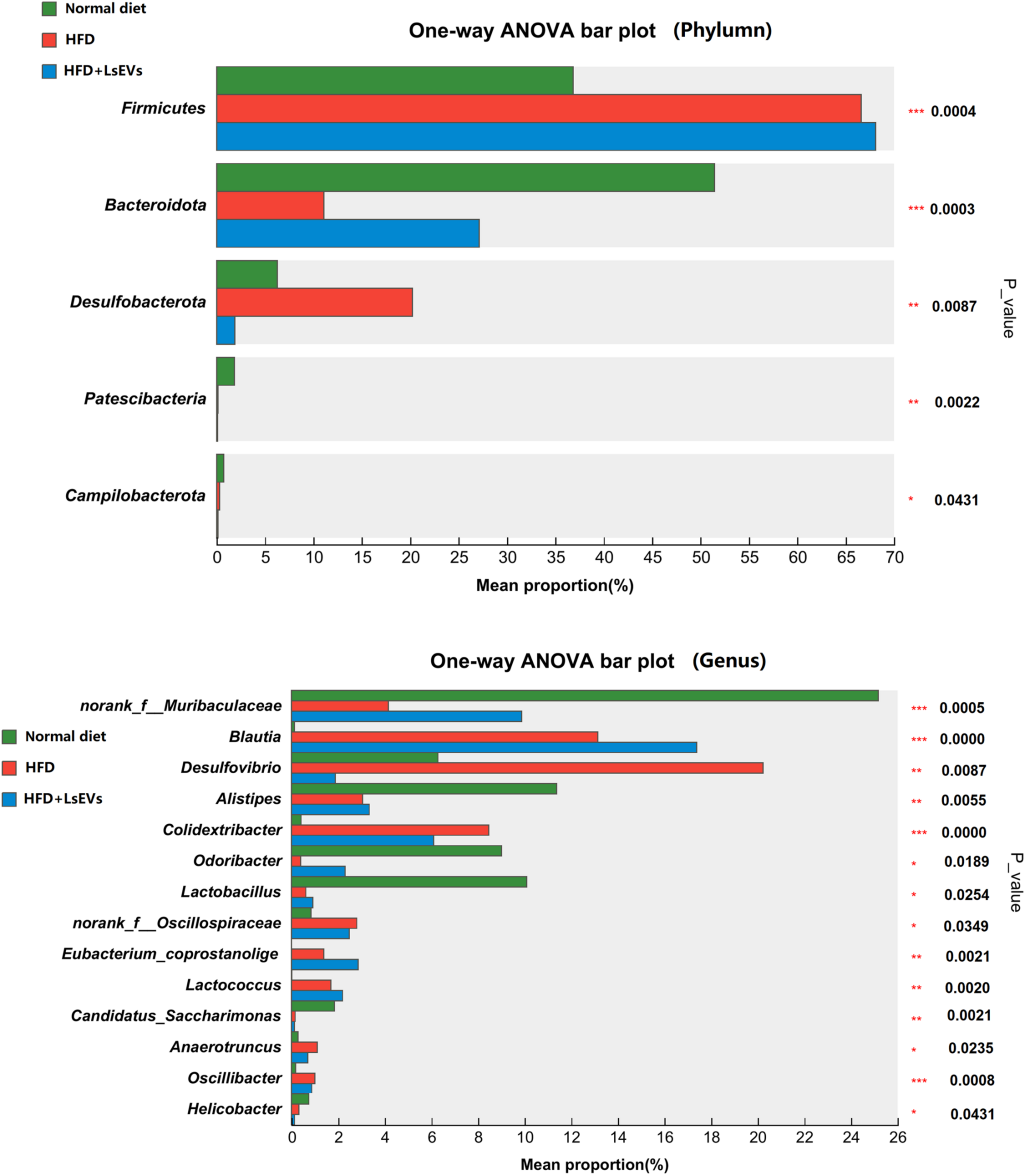
Differences in intestinal microbial phyla and genera levels. *P < 0.05, **P < 0.01, and ***P < 0.001. HFD, high fat diet; SNK-6, *L. salivarius* SNK-6; LsEVs, *L. salivarius* SNK-6-derived extracellular vesicles.

The abundance of five phyla (*Fimicutes*, *Bacteroidota*, *Patescibacteria*, *Desulfobacterota*, and *Campilobacterota*) and 14 genera (*Muribaculaceae*, *Blautia*, Desulfovibrio, *Alistipes*, *Colidextribacter*, *Eubacterium*_*corprostanoligenes*_group, norank_f_*Oscillospiraceae*, *Lactobacillus*, *Lactococcus*, and *Candidatus*_*Saccharimonas*) was different in the ceca from mice fed normal, HFD, and LsEvs (*P* < 0.05, **Figures 9c**, **10**). For example, at the phylum level, *Fimicutes* had more than 1.8-fold abundance in HFD mice compared with normal mice (*P* < 0.01), while *Bacteroidota* were much less abundant in HFD mice compared with normal mice a (*P* < 0.01, 11.08% vs 51.46%). LsEVs treatment significantly increased the abundance of *Bacteroidota* in the ceca of HFD mice (*P* < 0.01, **Supplementary Figure S2**, 27.13% vs 11.08%). At the genus level, the abundance of *Desulfovibrio* in mice treated with LsEVs was over ten times lower than that in those on HFD (*P* < 0.05, **Supplementary Figure S3**, 1.88% vs 20.23%).

*Muribaculaceae*, *Alistipes*, *Lactobacillus* are much higher in the normal diet treated mice than those in the HFD treated mice, LsEVs treatment tend to increase the abundance of these microorganisms, while no significant difference was found. Overall, *Lactobacillus* was enriched in the control group, while *Desulfovibri*o were enriched in HFD mice. LsEVs treatment significantly upregulated the abundance of *Eubacterium-coprostagenes* (**Figure 10**).

## 4 Discussion

MAFLD is a common liver disorder, with a notable increase in its prevalence observed in recent years, especially among those suffering from obesity and metabolic syndrome (1). Research has shown a significant link between disturbances in gut microbiota and both the development and advancement of MAFLD (18). Probiotics have garnered considerable interest recently as a key strategy for modulating gut microbiota. While the efficacy of *L. salivarius* as a probiotic in the context of MAFLD has been established, the precise mechanisms by which it operates remain unclear (12). In this study, we employ the LsEVs released by *L. salivarius* SNK-6 as a basis to thoroughly examine the influence of LsEVs on the regulation of hepatic lipid droplet buildup and the integrity of the intestinal barrier. We demonstrated that the administration of LsEVs can significantly reduce hepatic lipid deposition in mice with MAFLD, enhance mitophagy in hepatocytes, restore fundamental intestinal functions, and stimulate the proliferation of intestinal probiotics.

Multiple randomized controlled trials have demonstrated that probiotics positively influence liver function in patients with MAFLD. For instance, a meta-analysis revealed that probiotic therapy significantly reduces liver enzyme levels, including ALT and AST, while also improving liver fat infiltration (4). Furthermore, another study indicated that probiotic supplementation can enhance patients’ blood lipid profiles by lowering T-CHO and TG levels (3). Consistent with these findings, our research showed that administering both *L. salivarius* SNK-6 and its LsEVs effectively reduced liver ALT and AST activity, as well as inhibited weight gain in mice, suggesting a potential regulatory role of LsEVs on liver function.

EVs are nanoparticles enclosed by membranes that are secreted by different types of cells. They carry a range of bioactive compounds, including proteins, lipids, and nucleic acids, which are crucial for intercellular communication and interactions (18). Research has demonstrated that EVs are crucial for the pathophysiology of liver diseases. They not only serve as intercellular messengers, transmitting information, but may also critical for the progression of MAFLD. EVs can influence liver health by modulating liver cell functionality and enhancing inflammatory reactions (21). In addition, EVs derived from probiotics may influence liver metabolism and inflammation indirectly through the enhancement of gut microbiota balance, potentially benefiting the onset and progression of MAFLD (15). Here, through both *in vivo* and *in vitro* research, we have established that LsEVs can lower the release of pro-inflammatory cytokines (such as TNF-α) in liver cells, concurrently promoting the synthesis of anti-inflammatory cytokines (IL-4). TNF-α, IL-6, and IL-4 are pivotal components of the immune system, with their expression regulation mechanisms being intricate and multifaceted. The expression of IL-6 is positively regulated by IL-1β and TNF-α, while being subject to negative feedback inhibition by glucocorticoid receptors (22, 23). The secretion of IL-6 is both time-dependent and cell-type specific, potentially varying with the timing of experimental interventions (24). In present study, LsEVs treatment inhibited the secretion of TNF-α and activated IL-4, while the expression of IL-6 remain unaffected, may be attributed to distinct signaling pathways and cellular microenvironments.

Mitophagy refers to a process within cells responsible for the degradation and recycling of cellular components. It involves the transport of proteins and organelles to lysosomes, where they undergo degradation. This well-preserved mechanism is essential for preserving cellular balance by eliminating damaged organelles and protein aggregates, thereby supporting bioenergetic equilibrium and the recycling of nutrients (25). Mitophagy is not merely a bulk degradation system; it also shows selectivity in removing particular substrates, such as misfolded proteins and impaired organelles (26). Mitophagy has been linked to the pathogens of various diseases, such as neurodegenerative conditions and cancer, where it can have both protective and detrimental effects depending on the cellular context (27, 28).

Key proteins involved in this mitophagy pathway include LC3, Beclin-1, and Parkin, each playing distinct yet interconnected roles in autophagy regulation and function. LC3 is a well-known marker of mitophagy and is essential for the formation of autophagosomes. It exists in two forms: LC3-I, which is cytosolic, and LC3-II, which is lipidated and associates with autophagosomal membranes. The transformation of LC3-I into LC3-II represents a vital stage in the formation of autophagosomes and is frequently utilized as a marker of autophagic activity (29). Evidences have demonstrated that selective receptors, such as p62, interact with LC3 to facilitate the degradation of specific cargoes, including damaged organelles and protein aggregates (30). Beclin-1 is another pivotal protein in the mitophagy pathway, acting as a key regulator of autophagosome formation. It interacts with various proteins to initiate the autophagic process, including the class III phosphatidylinositol 3-kinase complex, which is essential for the formation of the phagophore (31). Beclin-1 interacts with BCL-2, which is a protein that inhibits apoptosis, and is essential for regulating mitophagy; breaking this connection may facilitate mitophagy and improve cell survival (32). Parkin, an E3 ubiquitin ligase, is chiefly recognized for its involvement in mitophagy, a process that the selectively degrades impaired mitochondria. It is attracted to damaged mitochondria, where it tags mitochondrial proteins with ubiquitin, signaling them for degradation through mitophagy (33). This process is essential for maintaining mitochondrial quality and preventing cellular stress. Recent findings indicate that Parkin also interacts with other mitophagy-related proteins, such as LC3, to aid in the incorporation of damaged mitochondria into autophagosomes (34). In present study, LsEVs treated mouse liver and FFA induced hepatocyte lipid droplet overload models both showed upregulation of autophagic pathway key markers including *Beclin-1*, *Parkin*, and *LC3* expression. The phenomenon of enhanced mitophagy was also observed by TEM and immunofluorescence analysis. Additionally, the expression of various receptors associated with the uptake of lipoproteins and FFAs was modified. The nuclear receptors, implicated in the fatty acid oxidation exhibited the following trends: *PPARγ* and *PPARα* mRNA levels were decreased in the HFD mice, while they were increased in *L. salivarius* SNK-6 and LsEVs treated mice. There was an increase in the expression of both *SREBP1* and *FASN* in the HFD group. Conversely, in mice treated with LsEVs, the level of *FASN* expression was noticeably decreased. All these indicating that LsEVs exert inhibitory effects on liver fat deposition may be through promoting fatty acid oxidation and mitophagy in liver cells and regulating the Beclin-1 related pathway.

The intestinal barrier function of fatty liver mice may be impaired, leading to increased intestinal permeability, which is known as microbiota translocation. This allows bacterial metabolites and components to access the liver via the portal vein, triggering inflammatory responses, oxidative stress, and lipid buildup, which may eventually result in fatty liver damage and liver fibrosis (35). Probiotic EVs play a vital role in maintaining the structural integrity of the intestinal epithelium (35). This integrity is a critical component in preventing the translocation of harmful pathogens and toxins from the gastrointestinal tract into the bloodstream. Numerous studies have demonstrated that these probiotic-derived EVs are capable of influencing the expression levels of essential tight junction proteins, including claudin-1 and ZO-1 (36). By enhancing the expression of these proteins, probiotic EVs contribute to the strengthening of the epithelial barrier, which is essential for safeguarding the body against various health threats (37). For example, research has demonstrated that treatment with *Lactobacillus plantarum*-derived EVs significantly increases these proteins levels, leading to improved transepithelial electrical resistance and reduced permeability of the intestinal barrier (36, 37). The improvement in barrier function is crucial for avoiding issues like inflammatory bowel disease and various gastrointestinal disorders, which frequently occur alongside weakened intestinal integrity (38, 39). Moreover, the anti-inflammatory properties of probiotic EVs contribute to their protective effects on the intestinal barrier. By inhibiting pro-inflammatory cytokines and modulating pathways such as NF-κB, these EVs can further support the maintenance of tight junction integrity under inflammatory conditions (40, 41). This dual action of enhancing tight junction protein expression while simultaneously reducing inflammation underscores the potential use of probiotic EVs in managing gut health and preventing intestinal barrier dysfunction. Here, in LPS induced intestinal epithelial cells, LsEVs treatment significantly inhibited the release of pro-inflammatory cytokines. In addition, we observed that oral administration of LsEVs can effectively repair the basic results of intestinal villi in HFD mice, modulating tight junction protein claudin-1, indicating the intestinal repair function of LsEVs.

The modulation of gut microbiota by probiotics is crucial for maintaining intestinal health, as dysbiosis can lead to various gastrointestinal disorders. Research indicates that *Lactobacillus* and *Bifidobacterium*, can improve the variety and quantity of beneficial bacteria while inhibiting harmful types, thus helping to restore microbial equilibrium and the integrity of the intestinal barrier (42–44). Probiotics may result in the formation of short-chain fatty acids (SCFAs), which are essential for preserving gut barrier integrity and influencing immune responses (45, 46). Including probiotics in dietary plans has demonstrated potential in enhancing intestinal health, highlighting the possible role as therapeutic agents for various gastrointestinal diseases (44, 47). Research has demonstrated that EVs derived from *Lactobacillus* and *Bifidobacterium*, can enhance host health by modulating the gut microbiota. Specifically, EVs from lactic acid bacteria have been shown to alleviate ulcerative colitis by influencing the polarization state of macrophages and maintaining intestinal homeostasis (48, 49). These vesicles have the ability to prompt macrophages to shift toward the M2 phenotype, promote the release of anti-inflammatory factors, and inhibit the production of pro-inflammatory factors, thus aiding in the restoration of intestinal barrier function and the modulation of the intestinal microbiota composition (48). Here, our results revealed that oral administration of LsEVs significantly changed the composition of microbiota in mice on a high-fat diet. The predominant bacterial phyla identified in the gut were *Bacteroidetes* and *Firmicutes*, which serve various crucial functions in gut health. These two phyla are capable of generating SCFAs, including butyric acid, propionic acid, and acetic acid, through the fermentation of dietary fibers. These SCFAs have an impact on the host’s energy homeostasis, obesity, inflammation, blood glucose regulation, insulin sensitivity, and hormone secretion (50). *Bacteroidetes* can activate T cell-mediated immune responses, which contribute to the overall health of the host. In addition, an increase in the proportion of *Firmicutes*/*Bacteroidetes* in obese individuals may be associated with increased calorie extraction from food, fat deposition, and impaired insulin sensitivity (51). We found that the proportion of *Firmicutes*/*Bacteroidetes* was increased in HFD mice, while decreased in LsEVs treated mice, also confirmed the beneficial effect of LsEVs in fat deposition regulation.

Notably, *Desulfovibrio*, also known as sulfate reducing bacteria, belongs to the phylum *Proteobacteria*. An important characteristic of *Desulfovibrio* is that they can produce hydrogen sulfide by “breathing” sulfates instead of oxygen (52). The production of hydrogen sulfide is believed to be toxic to intestinal epithelial cells and may lead to gastrointestinal diseases. In terms of human health, *Desulfovibrio* is associated with various diseases. They are associated with colorectal cancer, systemic sclerosis, gestational diabetes, constipation irritable bowel syndrome, bacteremia and other diseases. Here, the abundance of *Desulfovibrio* in HFD mice was significantly increased, but feeding LsEVs significantly reduced the abundance of *Desulfovibrio* in the gut, indicating that LsEVs regulate the intestinal barrier function of HFD mice, possibly by promoting the increase of *Bacteroidetes* while suppressing the growth of detrimental bacteria such as *Desulfovibrio*.

**Figure 11:**
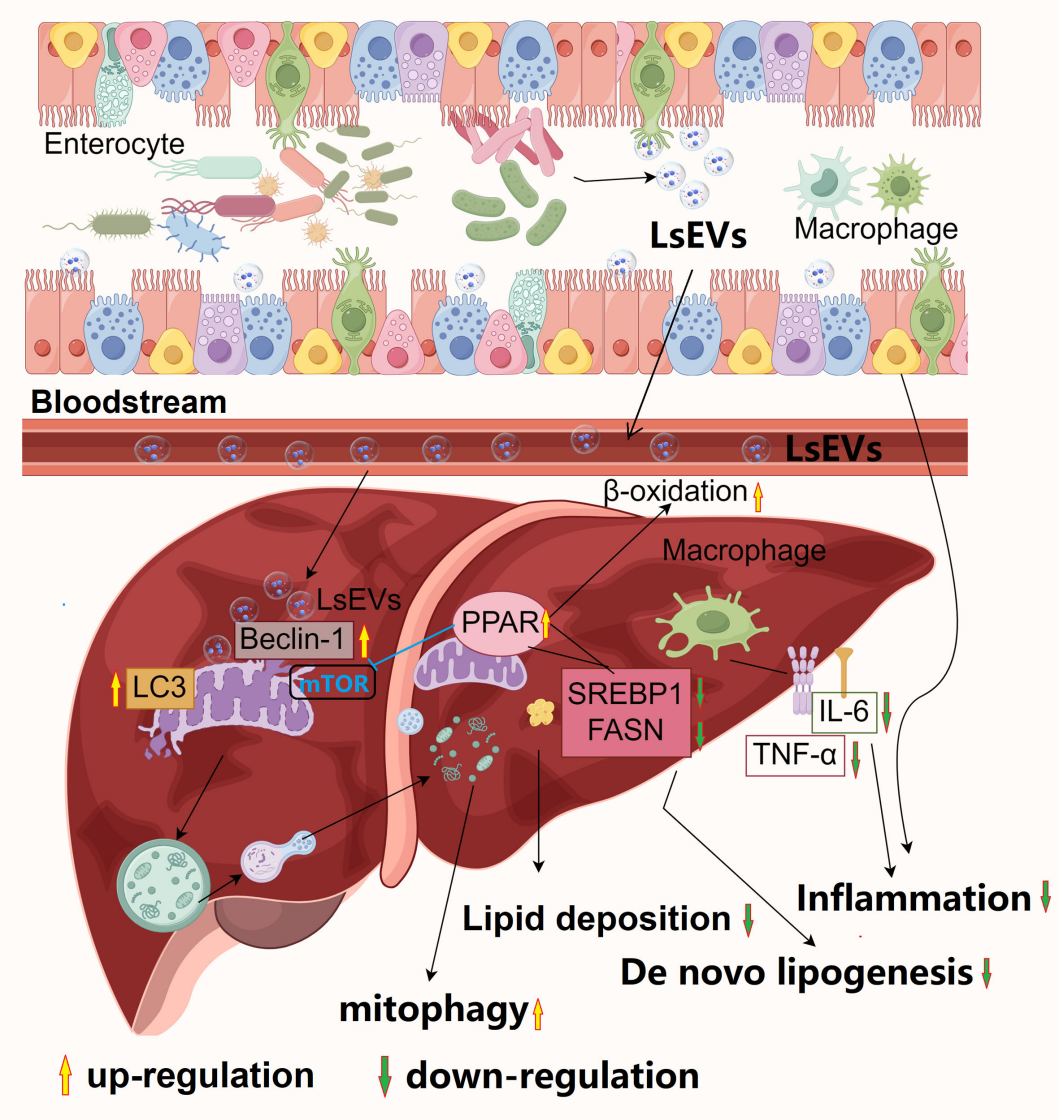
Proposed model for the beneficial effects of *L. salivarius SNK-6* on n MAFLD. *L. salivarius* SNK-6 secrets LsEVs, which can be observed by host cells, LsEVs increased the expression of Beclin-1 and LC3, which were found on the impaired mitochondria, simultaneously boosting autophagic flux. As a result, the damaged mitochondria were encapsulated by mitophagosomes and subsequently degraded by mitophagolysosomes. Meanwhile, the activation of β-oxidation occurred through the upregulation of PPARγ expression, which in turn suppressed *de novo* lipogenesis, leading to the improvement of hepatic steatosis, oxidative stress, and the activation of the inflammasome. Significantly, LsEVs contribute to the restoration of impaired intestinal barrier function and modulate the diversity of gut microbiota, promoting immune homeostasis in the intestines to counteract inflammation triggered by a high-fat diet in a mouse model. The picture was draw By Figdraw 2.0 (China).

The current research identifies the processes and roles of LsEVs (**Figure 11**). LsEVs is crucial in regulating fat deposition and mitochondrial autophagy, and it induces mitophagy in HFD induced mice. Observational results indicate that LsEVs treatment enhances liver autophagy in HFD mice, as evidenced by increased protein expression of Beclin-1 and LC3, alongside activation of the PPAR pathway. Furthermore, LsEVs effectively repair intestinal barrier dysfunction caused by HFD, suppress the release of inflammatory cytokines, promote the proliferation of *Bacteroidota*, and inhibit the growth of harmful bacteria *Desulfovibrio*.

In this study, we observed that both *L. salivarius* SNK-6 and LsEVs exhibit significant efficacy in mitigating fatty liver. However, it is important to highlight that, unlike probiotics, the efficacy of probiotics is often contingent upon specific strains, and the administration of live bacteria may present risks to immunocompromised individuals (53). Conversely, the utilization of probiotic-derived EVs in therapeutic contexts offers distinct advantages. Empirical studies have demonstrated that these vesicles possess the capability to traverse biological barriers, such as the blood-brain barrier, thereby contributing to the treatment of neurodegenerative disorders (54, 55). These vesicles not only encapsulate various bioactive molecules but can also be engineered for applications in drug delivery and vaccine development (56, 57). Consequently, future research should aim to further investigate the synergistic effects of these two approaches in MAFLD treatment to develop more effective therapeutic strategies. By integrating the biological activity of *L. salivarius* SNK-6 with the delivery capabilities of LsEVs, this approach may offer an innovative strategy for MAFLD treatment and advance the field of health science.

This study has three main limitations. First, the optimal dosage and duration of *L. salivarius* SNK-6 and LsEVs for treating MAFLD are unclear, requiring further research into their dose-response and timing. Second, the isolation methods for LsEVs, like ultracentrifugation and magnetic bead sorting, may alter their composition, affecting their efficacy in MAFLD regulation. Current research lacks systematic comparisons of these methods and their impact on vesicle function. Future studies should focus on dose gradients, time-course analyses, and method comparisons to improve understanding of probiotic vesicles in MAFLD treatment. Finally, research has demonstrated that EVs secreted by probiotics, play a significant role in host-microbe interactions, particularly in modulating immune responses and maintaining host homeostasis. These probiotic EVs are capable of transporting bioactive molecules, including proteins, lipids, nucleic acids, and metabolites, thereby facilitating complex signaling and substance exchange with host cells (58, 59). In our preliminary investigations, we have also identified the presence of multiple functional proteins within LsEVs, including certain immune regulatory factors, which can be internalized by host cells to modulate their immune function (17). Nevertheless, further research is required to elucidate which specific proteins within LsEVs are responsible for exerting probiotic regulatory effects.

In summary, LsEVs induces mitophagy in fatty liver through the involvement of the Beclin/PPAR pathway. These significant findings suggest that LsEVs could function as a therapeutic agent or system for the prevention of MAFLD. However, additional research with longer durations and different doses is essential to expand upon our present conclusions and to clarify the specific molecular pathways through which LsEVs promote autophagy flux in liver cells. This will improve our understanding of the potential therapeutic options of *Lactobacillus salivarius* and its vesicular LsEVs as preventive and/or therapeutic strategies for MAFLD.

## Data Availability

The datasets presented in this study can be found in online repositories. The names of the repository/repositories and accession number(s) can be found in the article/**Supplementary Material**.
