# Supplementary material for: Lactobacillus salivarius extracellular vesicles enhance gut and liver function in MAFLD
Source: Front Immunol. 2025 Aug 28;16:1660131. doi: 10.3389/fimmu.2025.1660131 (PMC12423052; doi:10.3389/fimmu.2025.1660131)
Supplement: Supplementary file 1 [file Table1.docx]

**Supporting information**

***Lactobacillus salivarius* Extracellular Vesicles Enhance Gut and Liver Function in MAFLD**

Lihui Zhu^1^, Jiwen Huang^1^, Zhen Luo^2^, Huaxiang Yan^1^, Xiao Wu^3*^, Rongrong Liao^1*^

^1^ Institute of Animal Husbandry and Veterinary Science, Shanghai Academy of Agricultural Sciences, Shanghai 201106, China.

^2^ Shanghai Key Laboratory of Veterinary Biotechnology, School of Agriculture and Biology, Shanghai Jiao Tong University, Shanghai 200240, China.

^3^ Key Laboratory of Agricultural Genetics and Breeding, Biotechnology Research Institute, Shanghai Academy of Agricultural Sciences, Shanghai 201106, China.

* To whom correspondence may be addressed:

**Xiao Wu**, Ph.D.

Key Laboratory of Agricultural Genetics and Breeding, Biotechnology Research Institute, Shanghai Academy of Agricultural Sciences, Shanghai 201106, China. Email: wuxiao@saas.sh.cn.

**Rongrong Liao**, Ph.D.

Institute of Animal Husbandry and Veterinary Science, Shanghai Academy of Agricultural Sciences.

Address: 2901 Beidi Road, Minhang distinct, Shanghai 201106, China. Email: liaorongrong@saas.sh.cn.

TEL: 86-021-62205472, Fax: 86-021-62205472.

**Table S1. List of primer sequences used for RT-PCR analysis in this study.**

| **Gene name** | **Former Primer（5'-3'）** | **Reverse Primer（5'-3'）** |
| --- | --- | --- |
| *LC3II* | TTATAGAGCGATACAAGGGGGAG | CGCCGTCTGATTATCTTGATGAG |
| *Parkin* | GAGGTCGATTCTGACACCAGC | CCGGCAAAAA TCACACGCAG |
| *FUNDC1* | AGAAGGTTGGAAAACTTGCTG | AGGTGCTGCTTTATTTGCTC |
| *BNIP3* | ACTCAGATTGGATATGGGATTG | GAAGGTGCTAGTGGAAGTTGTC |
| *Beclin1* | GATTGGACCAGGAGGAAGC | CTGTGCCAGATGTGGAAGG |
| *PINK1* | TGGAGGATTATCTGATAGGGC | TCTTGATGGCAAAGGGAAA |
| *PPARγ* | TAGGTGTGATCTTAACTGCCG | GCATCGTGTAGATGATCTCA |
| *SREBP1* | CGGAAGCTGTCGGGGTAG | GTTGTTGATGAGCTGGAGCA |
| *PPARα* | ACTGGTAGTCTGCAAAACCAAA | AGAGCCCCATCTGTCCTCTC |
| *FASN* | GGAGGTGGTGATAGCCGGTAT | TGGGTAATCCATAGAGCCCAG |
| *β-actin* | CCCATCTATGAGGGTTACGC | TTTAATGTCACGCACGATTTC |
| *GAPDH* | GGTGAAGGTCGGTGTGAACG | CTCGCTCCTGGAAGATGGTG |


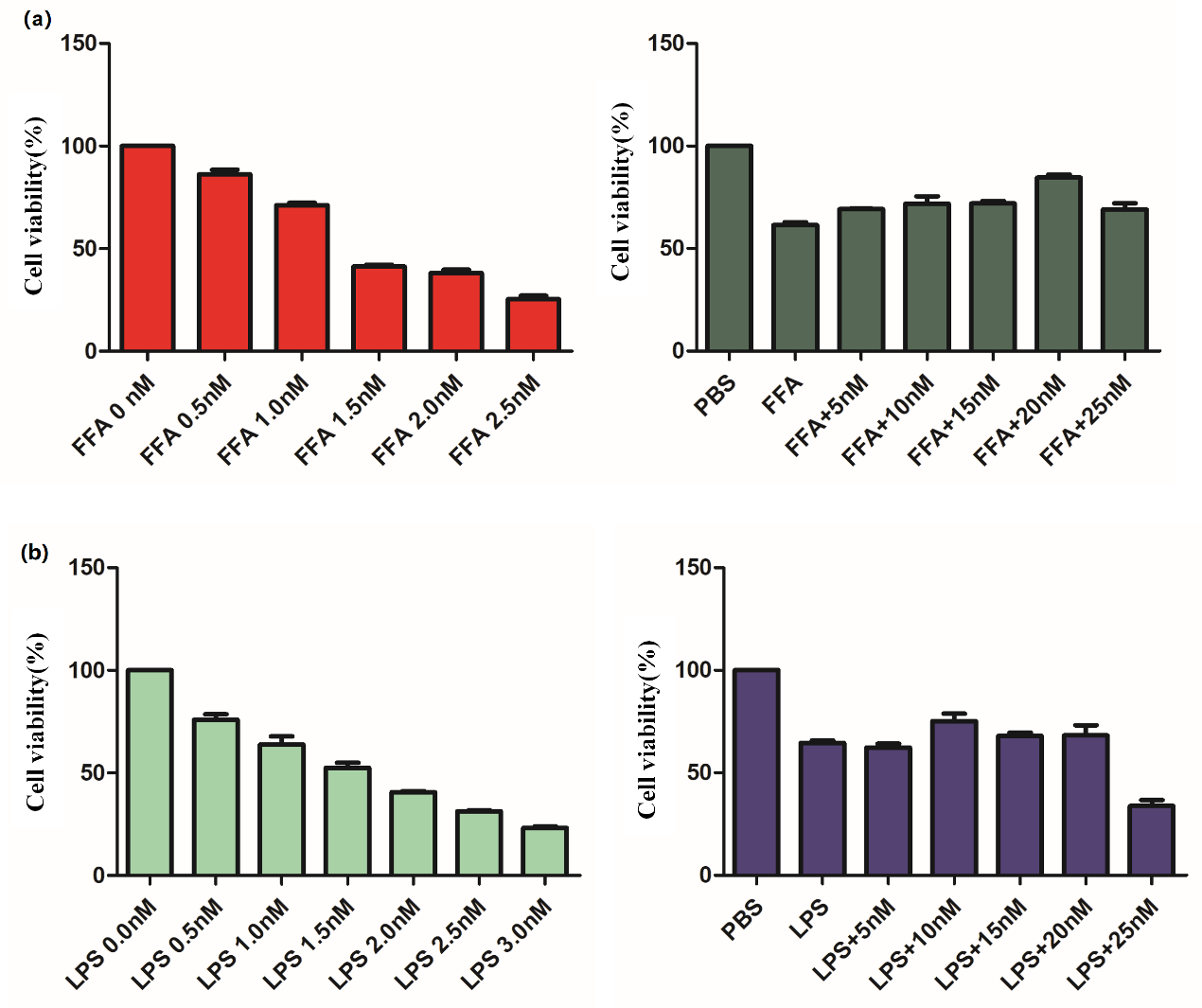


**Figure S1 Cell viability of LsEVs treated cells (a) NCTC1469 cells (b) Caco2 cells**


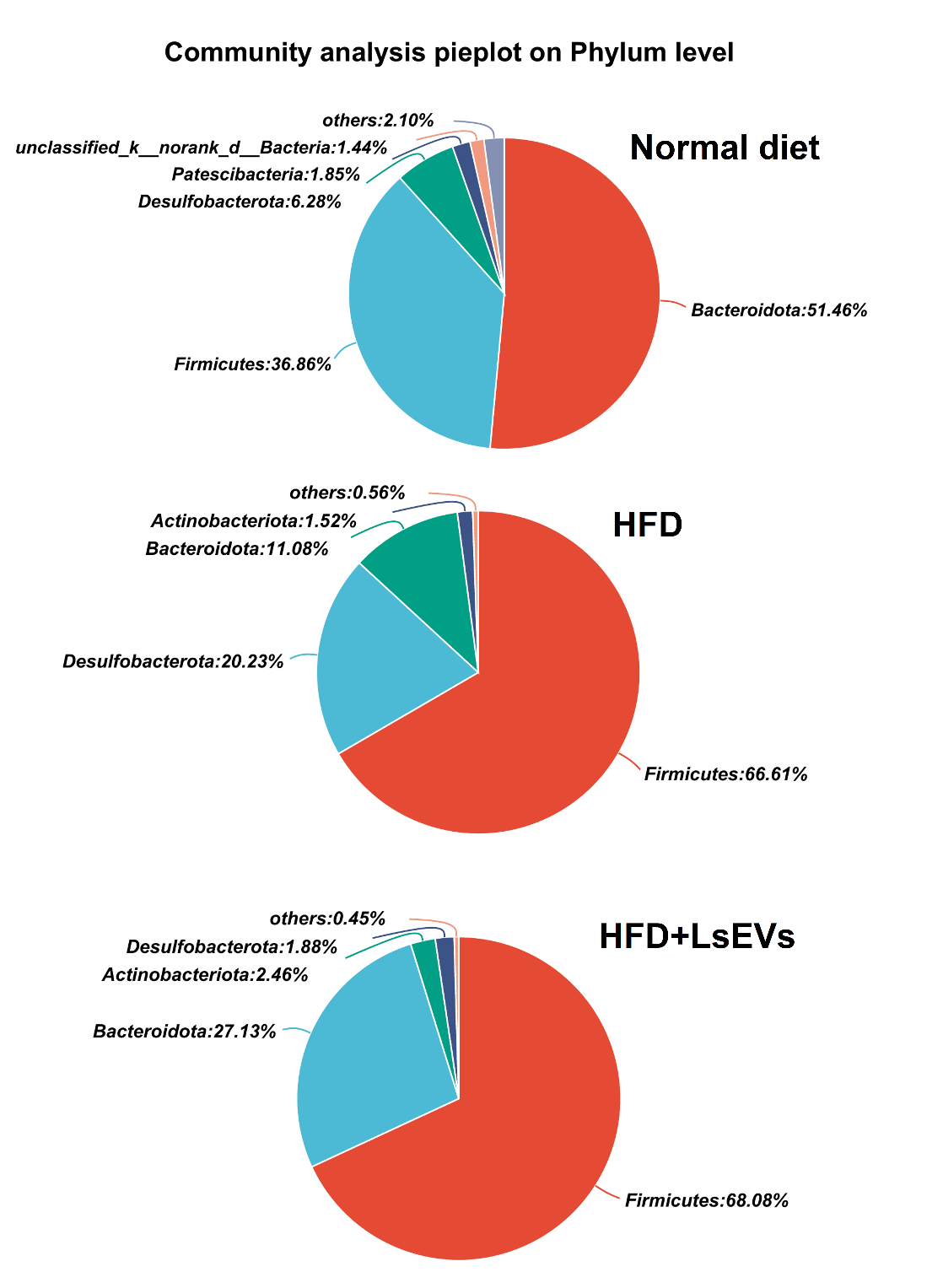


**Figure S2 Effects of LsEVs on gut microbiota at the phylum level in mice with fatty liver.**


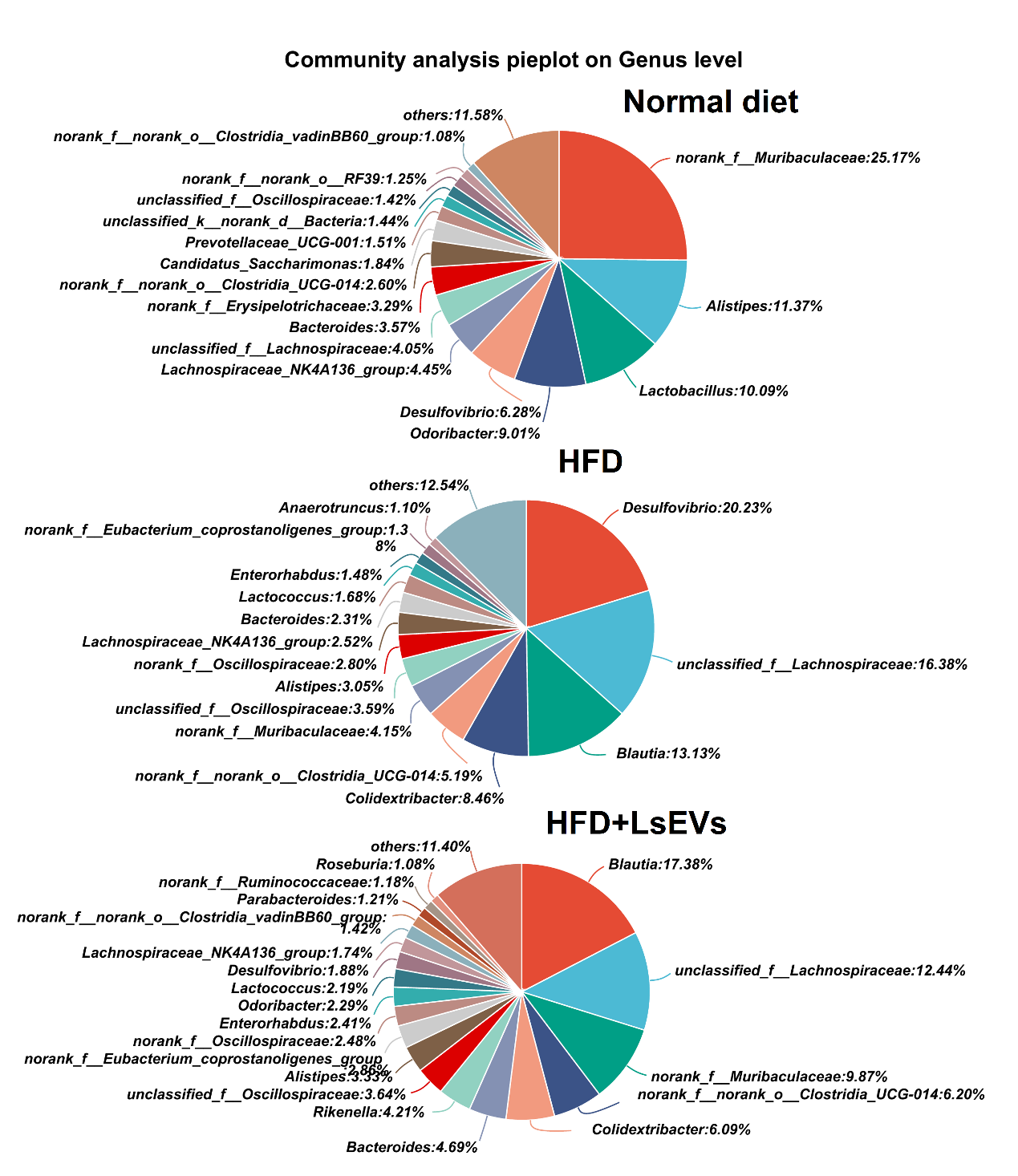


**Figure S3 Effects of LsEVs on gut microbiota at the genus level in mice with fatty liver.**
